# Biologic therapies for refractory juvenile dermatomyositis: five years of experience of the Childhood Arthritis and Rheumatology Research Alliance in North America

**DOI:** 10.1186/s12969-017-0174-0

**Published:** 2017-06-13

**Authors:** CH Spencer, K Rouster-Stevens, H Gewanter, G Syverson, R Modica, K Schmidt, H Emery, C Wallace, S Grevich, K Nanda, YD Zhao, S Shenoi, S Tarvin, S Hong, C Lindsley, JE Weiss, M Passo, K Ede, A Brown, K Ardalan, W Bernal, ML Stoll, B Lang, R Carrasco, C Agaiar, L Feller, H Bukulmez, R Vehe, H Kim, H Schmeling, D Gerstbacher, M Hoeltzel, B Eberhard, R Sundel, S Kim, AM Huber, A Patwardhan, Pediatric Rheumatologist Collaborators, Leslie Abramson, Dania Basodan, Johanna Chang, Megan Curran, Kyla Driest, Polly Ferguson, Daniel Horton, Kristin Houghton, Maria Ibarra, Esra Meidan, Marc Natter, Miriam Parsa, Reshma Patel, Sarah Ringold, Tova Ronis, Ken Schikler, Bacha Shaham, Elizabeth Stringer, Hemalatha Srinivasahu, Cagri Toruner, Mary Toth, Dawn Wahezi

**Affiliations:** 1 Nationwide Children’s Hospital and Ohio State University, Columbus, OH USA; 2 Emory University School of Medicine, Atlanta, GA USA; 3 Pediatric and Adolescent Health Partners, Richmond, VA USA; 4 University of Wisconsin-Madison, Madison, WI USA; 5 University of Florida, Gainesville, FL USA; 6 University of Louisville, Louisville, KY USA; 7 Seattle Children’s Hospital, University of Washington, Seattle, WA USA; 8 Riley Hospital for Children, Indiana University Medical Center, Indianapolis, IN India; 9 University of Iowa Health Care, Iowa City, IA USA; 10 University of Kansas Medical Center, Kansas City, KS USA; 11 Sanzari Children’s Hospital, Hackensack University Medical Center, Hackensack, NJ USA; 12 University of South Carolina, Charleston, SC USA; 13 Phoenix Children’s Hospital, Phoenix, AZ USA; 14 Texas Children’s Hospital, Houston, TX USA; 15 Lurie Children’s Hospital, Chicago, IL USA; 16 University of California, San Francisco, San Francisco, CA USA; 17 University of Alabama at Birmingham, Birmingham, AL USA; 18 WK Health Center and Dalhousie University, Halifax, NS Canada; 19 Dell Children’s Hospital, Austin, TX USA; 20 Children’s Hospital of The Kings Daughter, Norfolk, VA USA; 21 Inland Rheumatology, Waterville, ME USA; 22 Metro Health Medical Center and Case Western Reserve University, Cleveland, OH USA; 23 University of Minnesota, Minneapolis, MN USA; 24 National Institute of Arthritis and Musculoskeletal and Skin Diseases, National Institutes of Health, Bethesda, MD USA; 25 Alberta Children’s Hospital, Cumming School of Medicine, University of Calgary, Calgary, AB Canada; 26 Lucille Packard Children’s Hospital, Stanford University, Stanford, CA USA; 27 Mott Children’s Hospital, University of Michigan, Ann Arbor, MI USA; 28 Cohen Children’s Medical Center of New York, New york, USA; 29 Boston Children’s Hospital and Harvard Medical School, Boston, MA USA; 30 School of Medicine, University of Missouri, Columbia, MO USA

## Abstract

**Background:**

The prognosis of children with juvenile dermatomyositis (JDM) has improved remarkably since the 1960’s with the use of corticosteroid and immunosuppressive therapy. Yet there remain a minority of children who have refractory disease. Since 2003 the sporadic use of biologics (genetically-engineered proteins that usually are derived from human genes) for inflammatory myositis has been reported. In 2011–2016 we investigated our collective experience of biologics in JDM through the Childhood Arthritis and Rheumatology Research Alliance (CARRA).

**Methods:**

The JDM biologic study group developed a survey on the CARRA member experience using biologics for Juvenile DM utilizing Delphi consensus methods in 2011–2012. The survey was completed online by the CARRA members interested in JDM in 2012. A second survey was similarly developed that provided more opportunity to describe their experiences with biologics in JDM in detail and was completed by CARRA members in Feb 2013. During three CARRA meetings in 2013–2015, nominal group techniques were used for achieving consensus on the current choices of biologic drugs. A final survey was performed at the 2016 CARRA meeting.

**Results:**

One hundred and five of a potential 231 pediatric rheumatologists (42%) responded to the first survey in 2012. Thirty-five of 90 had never used a biologic for Juvenile DM at that time. Fifty-five of 91 (denominators vary) had used biologics for JDM in their practice with 32%, 5%, and 4% using rituximab, etanercept, and infliximab, respectively, and 17% having used more than one of the three drugs. Ten percent used a biologic as monotherapy, 19% a biologic in combination with methotrexate (mtx), 52% a biologic in combination with mtx and corticosteroids, 42% a combination of a biologic, mtx, corticosteroids (steroids), and an immunosuppressive drug, and 43% a combination of a biologic, IVIG and mtx. The results of the second survey supported these findings in considerably more detail with multiple combinations of drugs used with biologics and supported the use of rituximab, abatacept, anti-TNFα drugs, and tocilizumab in that order. One hundred percent recommended that CARRA continue studying biologics for JDM. The CARRA meeting survey in 2016 again supported the study and use of these four biologic drug groups.

**Conclusions:**

Our CARRA JDM biologic work group developed and performed three surveys demonstrating that pediatric rheumatologists in North America have been using multiple biologics for refractory JDM in numerous scenarios from 2011 to 2016. These survey results and our consensus meetings determined our choice of four biologic therapies (rituximab, abatacept, tocilizumab and anti-TNFα drugs) to consider for refractory JDM treatment when indicated and to evaluate for comparative effectiveness and safety in the future.

Significance and Innovations

- This is the first report that provides a substantial clinical experience of a large group of pediatric rheumatologists with biologics for refractory JDM over five years.
- This experience with biologic therapies for refractory JDM may aid pediatric rheumatologists in the current treatment of these children and form a basis for further clinical research into the comparative effectiveness and safety of biologics for refractory JDM.

**Electronic supplementary material:**

The online version of this article (doi:10.1186/s12969-017-0174-0) contains supplementary material, which is available to authorized users.

## Background

Juvenile dermatomyositis (JDM) is a systemic autoimmune disease in children and adolescents characterized by a vasculopathy that primarily affects skin and muscle but may involve the lungs, heart, gastrointestinal system, joints, and other organs. Before the consistent use of daily corticosteroids in the 1950’s, 1/3 of JDM children had a fatal outcome, 1/3 became disabled, and 1/3 recovered [1, 2]. Since then the prognosis has improved significantly with less than 1% mortality with current early diagnosis and aggressive therapy [3–14]. Complications are still not uncommon, including calcinosis, contractures, vasculitis, and lipodystrophy. Treatment may lead to side effects, especially due to corticosteroids. Also, JDM treatment has varied tremendously among rheumatologists and other specialists with the use of oral and intravenous corticosteroids, hydroxychloroquine, and immunosuppressive drugs such as methotrexate (mtx), cyclosporine (CSA), azathioprine (AZA), mycophenolate (MMF), and cyclophosphamide (CYC) [15–23]. Despite these aggressive treatments, a significant minority of children with JDM have a difficult clinical course, even life-threatening [4–14].

There has been increasing evidence documenting the critical role of cytokines as regulators of inflammation in inflammatory myopathies. Tumor necrosis factors (TNFα, LTβ, BAFF) interferons (IFNα/β/ϒ), interleukins (IL-1, IL-6, IL-12, IL-15, IL-18, IL-23), and chemokines (CXCL9/10/11/13, CCL2/3/4/8/19/21) have been reported to be have a role in these muscle diseases [24]. The use of biologic agents targeting these cytokines is now prevalent in the treatment of numerous childhood autoimmune conditions, especially for juvenile idiopathic arthritis [25, 26].

Due to the above morbidity and the new science, the use of these biologic medications for myositis has begun on an off-label basis since 2000 in numerous countries. The most commonly used biologic in the last decade for inflammatory myositis appears to be rituximab. The Rituximab in Myositis (RIM) study of rituximab effectiveness in adult and pediatric myositis did not meet its primary endpoint for JDM, though the study did show that 83% of the adult and pediatric myositis patients met the definition of improvement on rituximab. Further analysis of the RIM data may allow approval of rituximab in the future [27, 28]. Infliximab, an anti-TNF-α medication, has also shown considerable potential for juvenile and adult myositis [29–32]. In contrast, another anti-TNFα drug, etanercept, does not appear to be as effective for myositis [33–35]. The use of a third anti-TNF-α drug, adalimumab, has not yet been reported in myositis treatment but has been helpful for interstitial pneumonitis associated with adult dermatomyositis, anti-synthetase syndrome, and orbital myositis [36–38]. Also abatacept, an anti-CTAL-4 monoclonal, has been beneficial in an adult overlap myositis resistant to other treatment [39], in refractory polymyositis [40], and in a recalcitrant JDM child with ulcerations and calcinosis [41]. The anti-IL-6 drug tocilizumab has shown potential for treatment of myositis as it has in other rheumatic diseases [42–44]. Other potential biologics for inflammatory myositis are sifalimumab (anti-IFNα), alemtuzumab (anti-CD52), eculizumab (a terminal complement inhibitor) and basiliximab (anti-CD25), but none has not been used much in children as of 2017 [45].

We believed that there was ample support in the medical literature for use and study of biologics off label for refractory JDM. The goal of this article is to report the results of a project performed by our North American Childhood Arthritis and Rheumatology Research Alliance (CARRA) research committee on biologics for refractory JDM.

## Methods

### Carra

CARRA is an organization of pediatric rheumatologists, researchers, and other interested parties in North America that was formed in 2002. Its mission is to prevent, treat, and cure rheumatic diseases of children and adolescents by facilitating and conducting high-quality clinical, translational, and bench research. By 2016 CARRA has grown to have over 400 members in 80 centers in the United States and Canada.

### Surveys

Two surveys on the use of biologics for JDM were developed in 2011–13 by consensus discussion of the Juvenile DM biologic committee by e-mail. The first survey of 15 questions that was sent out in February–March 2012 was a general survey on biologics with limited choices for respondents (Additional file 1: Appendix A). It was sent to the general CARRA membership at the time. The results of the first survey are in the results section. A second survey was developed and completed in 2013. The second survey focused on more specifics of the use of biologics with the opportunity for extended comments and on details of the future CARRA studies (inclusion and exclusion criteria, testing, and outcome measures-results not reported here). The second survey results are in Additional file 1: Appendix C. Thirty pediatric rheumatologists who attended JDM Committee meetings at the 2016 CARRA meetings ranked the biologics anonymously using the rank choice technique to check for any change of opinion on the use of biologics for JDM.

### Ethics approval and consent to participate

The authors asked for and received Institutional Review Board approval with a waiver from the Nationwide Children’s Hospital IRB (2012) and the University of Missouri Medical Center IRB (2013) for survey #1 and survey #2 respectively. No consents were justified.

### Consensus meetings

Consensus meetings on biologics were held at five annual CARRA meetings from 2011 to 2016. Each 3–5 h meeting consisted of a core group of pediatric rheumatologists and researchers with a maximum of 30 participants per meeting. After developing the two biologic surveys in 2011–2013, the 2013–16 CARRA meetings were used to discuss biologics for refractory JDM as reported in the literature [27–44] and beginning discussions of a future consensus treatment plan. The nominal group technique was utilized to achieve consensus with one facilitator (AP) and one recorder (CHS) at JDM biologic workgroup sessions during the 2013, 2014, and 2015 CARRA meetings. The identical format was used for each question. If one response eventually won over 80% of the votes, that response was chosen. This process was continued until each question posed had achieved consensus. The 2015–16 sessions finalized the choice of the biologics. These discussions were informed by the three surveys, the experiences of participants, and the current medical literature, especially the rituximab in myositis (RIM) international study [27–44].

## Results

The first survey can be found in Additional file 1: Appendix A. The results were as follows: The survey was sent to the entire membership of CARRA in 2012 (250 members at the time) and 104 responded (42%). Ninety-seven (93.3%) of the 104 CARRA members replied that they were interested in biologics in JDM and 7/104 replied No (6.7%) and did not continue the survey. Different numbers of the potential 97 respondents answered every question varying from 40 to 91 based on their choice whether any one respondent wanted to answer that one question.

At the time of the survey, 79/95 (83%) were practicing pediatric rheumatologists. Nine were trainee fellows; five fellows were in years 1–2 and 4 in years 3–4. Five (5.3%) were medicine-pediatric rheumatologists, one was a non-practicing researcher pediatric rheumatologist (1.1%), and was one was a nurse practitioner (1/95–1.1%). The special rheumatology niche of the respondents included SLE (18/91–20%), JIA (22/91–24%), vasculitis (4/91–4%), myositis IIM (10/91–11%), scleroderma (2/91–2%), basic science research (5/91–6%), and nothing specific (24/91–26%). Six answered “other” (6/91–6%). It was an underlying assumption of the authors of the 2012 survey that any member of CARRA that responded were likely to have some training and practice in all diseases of pediatric rheumatology, including JDM, and were qualified to respond even if they had other special interests.

Fifty-one (56.7%) of ninety respondents currently managed 1–10 total children with JDM, 30/90 (33.3%) managed 11–20, 5/90 (5.6%) 20–50, and 4/90 (4.4%) managed over 50 patients. Eighty-five of 91 (93.4%) saw 1–10 new JDM patients per year, 3/91 (3.3%) saw 11–20 new patients per year, and 3 (3.3%) saw 20–50 per year.

Thirty-five practitioners had not yet used biologics for JDM in 2012. Fifty-five (61.5%) of 91 responding practitioners had used biologics in their practice with 32%, 5%, and 4% having used rituximab, etanercept, and infliximab respectively. Seventeen percent had used more than one of these biologics at separate times. One respondent (1%) answered “other” (Table 1).

**Table 1:** Experience with biologics in juvenile dermatomyositis in survey #1 in 2012

No experience at all	35 (39%)
Etanercept	5 (5%)
Abatacept	0 (0.0%)
Adalimumab	0 (0.0%)
Infliximab	4 (4%)
Anakinra	1 (1%)
Tocilizumab	0 (0.0%)
Rituximab	29 (32%)
Canakinumab	0 (0%)
Multiple	16 (18%)
Other	1 (1%)

The vast majority (90%) used biologics in combination with other medications at one time or another: mtx (11/57, 19%), mtx + steroids (30/57, 53%), mtx, steroids, and MMF or AZA (24/57 42%). Ten percent had used a biologic as a monotherapy. Nine respondents chose “other” (15.8%) (Table 2).

**Table 2:** Pediatric rheumatologists in the survey who have used biologics for JDM in combination with other therapies. Note-PR’s could answer more than once

Monotherapy of a biologic	6 PR’s
Biologic + methotrexate	11 PR
Biologic + methotrexate + corticosteroids	30 PR
Biologic + methotrexate + corticosteroids +1 immune-suppressive (MMF, AZA, or CSA)	24 PR
Biologic + methotrexate +IVIG	25 PR
Biologic + other therapies	9 PR

*mtx* oral, subcutaneous, or IV methotrexate, *CS* oral or intravenous corticosteroids, *AZA* azathioprine, *MMF* mycophenolate, *CSA* cyclosporine A

For those who did not use a biologic for JDM, the reasons varied considerably. Two of the 26 (9%) respondents answered that they did not believe biologics worked for JDM. Three of 26 (13%) respondents did not use biologics due to insurance denial. One of 26 (4%) PR did not use a biologic due to parent denial, 15/26 (64%) due to not being sure a biologic would work, and 2/26 (9%) due to cost of therapy.

Uncontrolled disease was the primary reason for use of a biologic (52/58, 89.7%). Steroid or mtx toxicity also was a rationale in 24/58 respondents (41.4%) and steroid dependence in 21/58 (36.2%). A family request was a factor in 6/58 (10.3%), multiple reasons in 13/58 (22.4%), and other reasons 8/58 (13.8%). The reasons many respondents did not answer this question are unclear.

The exact situation that the pediatric rheumatologists chose to start a biologic for JDM was queried (Table 3). Multiple answers were allowed: Five of 94 (5.3%) started a biologic after the child failed mtx only, 14/94 (14.9%) after failing mtx and steroids, 39/94 (41.5%) after failing steroids, mtx, as well as IVIG, and 48/94 (51.1%) after failing mtx, steroids, IVIG and an another immunosuppressant (AZA/MMF/or CSA). Ten of 94 (14.9%) respondents started a biologic for systemic JDM with internal organ involvement while fourteen of 94 (14.9%) chose to start a biologic for severe ulcerative disease of JDM. Twenty-seven of 94 (28.7%) started a biologic for other reasons than the responses allowed by the survey.

**Table 3:** The exact situations in which biologics were utilized for JDM. Respondents could answer more than one time. Ten potential respondents did not choose any of the choices

After failing mtx	5/94 5.3%
After failing CS and mtx	14/94 14.9%
After failing CS, mtx, and IVIG	39/94 41.5%
After failing CS, mtx, IVIG, or IM (AZA, MMF, or CSA)	48/94 51.1%
Systemic JDM with internal organ involvement	10/94 14.9%
Severe ulcerative disease	14/94 14.9%
Other	27/94 14.9%

*Abbreviations*: *mtx* oral, subcutaneous, or intravenous methotrexate, *CS* oral or intravenous corticosteroids, *AZA* azathioprine, *MMF* mycophenolate, *CSA* cyclosporine A

The respondents were also asked their opinion as to the overall effect of the use of a biologic. They could choose more than one option. The results were improvement 49/67 (73.1%), no change 13/67 (19.4%), less calcinosis 8/67 (11.9%), worsening of the disease 7/67 (10.4%), more side effects than improvement 6/67 (9.0%), less growth retardation 3/67 (4.5%), unable to access 9/67 (13.4%), or other effects 10/67 (14.9%).

Twenty-one of 40 (52.5%) reported that the complication of calcinosis was reduced on a biologic while 11/40 (27.5%) reported that muscle atrophy improved. Seven of 40 (17.5%) respondents noted that lipodystrophy was reduced while being treated with a biologic. Eight of 40 respondents (20%) also believed that osteonecrosis complications were reduced while their patients were on a biologic. Seventeen of 40 (42.5%) believed that a biologic helped the severe ulcerative disease of JDM and 10/40 (25%) the severe complications of internal organ perforation/necrosis/damage. Nineteen of 40 respondents (47.5%) noted other improvements in JDM complications that could not be listed due to the format of the survey (Table 4).

**Table 4:** In your experience, can the use of biologics in JDM patients reduce any of the following complications?

Calcinosis	21/40 52.5%
Muscle atrophy	11/40 27.5%
Lipodystrophy	7/40 17.5%
Osteonecrosis	8/40 20%
Ulcerative disease	17/40 42.5%
Internal organ damage/perforation/necrosis	10/40 25%
Other complications	19/40 47.5%

Twenty-three of 36 respondents (63.9%) noted rituximab side effects at some time while 6/36 respondents (16.7%) reported abatacept side effects. Six of 36 also had experience with side effects on etanercept. Four respondents 4/36 (11.1%) noted adalimumab side effects while 9/36 (25%) infliximab side effects. Five (13.9%) respondents recalled problems on anakinra while 2/36 (5.6%) noted tocilizumab side effects. Four (4/36, 9.9%) reported canakinumab side effects. Two respondents reported having multiple drug side effects and six (16.7%) reported other responses.

A majority of the CARRA members answering responded that if the opportunity arose, they would use a biologic for JDM (41/47, 87%) while only 3/47 (6%) said that they would not. (Note: this low denominator may indicate that not all respondents wished to express an opinion on this question). Seventy percent of the respondents (63/90) recommended that CARRA study biologics in Juvenile DM, 6% responded no (5/90), 23% (21/90) said they were not sure and 1/90 (1.1%) answered “other”. In contrast, 100% of respondents to Survey #2 recommended CARRA study biologics in JDM.

### Survey #2

The second survey’s purpose was the following: a) Obtain more details on the current and previous use of biologics for JDM by interested CARRA pediatric rheumatologists including the preferred biologics, rationales for their use, combinations of drugs used with the biologic, and to identify which exact biologic might be the primary or secondary drug to use (See Additional file 1: Appendix B); It was also designed to begin the development of the protocol for any future biologic CTP (consensus treatment plan-data not reported in this report).

Review of the results of this survey confirms the diverse and widespread use of biologics within the CARRA group. Rationales for use were documented (Additional file 1: Appendix C). There were no recognizable patterns of which biologics were added to which combination of corticosteroids, immunosuppressant’s, and IVIG and for what rationale. The results do show support for the use of rituximab, as well as adalimumab, infliximab, abatacept, tocilizumab, and even etanercept.

During the study group sessions of 2014–2015, the study group used these survey results, the current literature, their experiences, and discussion using the nominal group techniques to decide on the ranking of preferences for biologics for refractory JDM. The rankings for treatment and study in these sessions were rituximab, abatacept, infliximab or adalimumab, and tocilizumab in that order.

### Survey #3

At the April 2016 CARRA meeting, the 31 physicians attending the JDM work groups ranked the biologics they would use in 2016 for a refractory JDM child unresponsive to corticosteroids, methotrexate, and IVIG utilizing ranked-choice voting (choice 1 high, 5 low). No IRB approval was needed. Rituximab was ranked first (rank mean 1.2), with abatacept (rank mean 2.4) and tocilizumab (rank mean 2.5) ranked second and third, respectively. Infliximab was the fourth choice (rank mean 3.4) and adalimumab the fifth (rank mean 4.3). The preferences for which biologics to use for refractory JDM appeared to remain essentially unchanged from 2012 to 2016 except for tocilizumab moving to a higher ranking.

## Discussion

Children with JDM have an excellent prognosis on current treatment in 2016 [2]. Treatment with topical ointments, corticosteroids (prednisone, methylprednisolone), hydroxychloroquine, methotrexate, azathioprine, cyclosporine, and IVIG in different combination treatment regimens has made a tremendous difference. A minority of children with JDM continue to have a difficult course with complications [2]. This JDM group is the target of our efforts with a goal of optimizing our aggressive therapy.

Biologic therapies have revolutionized the treatment of chronic arthritis in children and adults since the late 1990’s with markedly improved outcomes and better function. This impressive leap in outcomes cannot be overemphasized [11–14]. These drugs have targeted pro-inflammatory cytokines and their receptors such as tumor necrosis factor, interleukin 1, and interleukin six. Other targets include the B cell antigen CD20 and the CTLA Ig molecule. Drugs that target other molecules such as CD-17, CD-23, CD-52 and other molecules are in development [43, 45]. Though these drugs are expensive, they appear to be more effective than any other arthritis and rheumatic disease treatment.

Biologic drugs have been used off-label since 2000 for JDM and other inflammatory myositis diseases with encouraging results published [25–45]. Rituximab has been used the most and is believed to have definite benefit [27, 28]. It was our belief that these biologic drugs might benefit children with refractory JDM and these drugs needed to be evaluated. No randomized clinical trials utilized to provide evidence-based guidelines that would improve clinical outcomes appear likely for this rare disease and trials on the use of one biologic do not help to distinguish the comparative effectiveness of different biologics. We began our surveys on biologics in JDM as a possible precursor to comparative effectiveness research approach (CER) used in our organization that might be able to compare different biologic treatments in a multicenter JDM trial.

Our study developed two surveys initially to capture the experience of our CARRA members with biologics in JDM. Both surveys had a respectable sample of pediatric rheumatologists in North America and notably included PR’s who care for a large number of children with JDM. The majority of the PR’s surveyed had used biologics for JDM as we suspected and the reasons why the minority had never used biologics for JDM were quite predictable. Most North American PR’s surveyed did not start treatment of JDM with biologic monotherapy but only used a biologic after steroids, methotrexate, IVIG or another immunosuppressive in some combination have been tried.

It appears important that the survey respondents believed that the biologics significantly reduced complications, particularly calcinosis, muscle atrophy, contractures, lipodystrophy, and osteonecrosis. Side effects were infrequent with patients on rituximab therapy having the most. The most telling result was that 73% of the respondents indicated that children with resistant JDM appeared to benefit from biologic treatment, supporting our belief that biologics were a logical therapeutic step after failure of corticosteroid, IVIG, and immunosuppressive therapy. Also, 87% respondents in the first survey favored using a biologic for JDM if the opportunity would arise. Finally, 70% of respondents in the first survey and 100% in the second survey recommended that CARRA study biologics in JDM.

The second survey results provides for interested pediatric rheumatologists more detailed and rich information on what pediatric rheumatologists were doing with these biologics for JDM as well as their 2013 preferences for the use of biologics for future study. These opinions provided a starting point for the discussions from 2013 to 2015 at our CARRA consensus meetings. It bears repeating that the involved PR’s reviewed the surveys, the medical literature, and their experience in making the choice of the four treatment arms studying biologics in JDM using the consensus methods in face-to-face meetings. Without explicit treatment guidelines, the treatments being used are empirical and vary tremendously rheumatologist-to-rheumatologist. This empirical approach, of course, provides no evidence-based consensus to decide which biologic treatment or treatments are optimal. The best solution may be future multicenter comparative effectiveness research to define guidelines for refractory JDM biologic treatments [46–50].

The third survey’s purpose was to continue to see if opinions of the CARRA JDM work group members had changed by 2016 on the biologics to use for refractory JDM. Thirty pediatric rheumatologists filled out the survey in April, 2016. A case was described of a child with JDM who was unresponsive to corticosteroids, methotrexate, and IVIG. The rheumatologists ranked their preferences of biologics using rank-choice voting of 1–5 (1 top preference) and chose rituximab with abatacept and tocilizumab next-no major change. Though etanercept received some votes, the consensus opinion of the group was that it is not likely to be effective for refractory JDM.

There are limitations to our survey approach. There was likely a responder bias as the pediatric rheumatologists who answered our surveys were likely the ones who had the most interest in JDM and biologic treatment for JDM. Other PR’s who did not respond to the surveys may have had other opinions that were not captured in these surveys. The surveys also had considerable variation in the number of answers of CARRA respondents to any particular question. This variability was likely due to differences in interest in the biologics and wide variation in experience with these drugs. There were no “hard stops” requiring respondents to answer any of the questions. Each CARRA member could choose which question to answer. The surveys also left out a question on the ability to reduce corticosteroid doses by use of the biologics. Although there were a large number of pediatric rheumatologists who responded to the survey and participated in our discussions, there were those in the groups who likely disagreed with the consensus decisions made. Also, these recommendations could not replace the clinical acumen and judgment of each rheumatologist facing the wide clinical variability of any one JDM patient with resistant disease. The process took four years as the CARRA JDM study group worked on the survey and consensus process as well as constructing a consensus treatment plan which delayed this report.

## Conclusions

Our CARRA biologic JDM work group is reporting the first experience of a broad cross-section of North American pediatric rheumatologists on the usefulness of biologics in refractory JDM. Three surveys demonstrated that a significant group of pediatric rheumatologists in the US and Canada are using different biologic therapies for this select group of refractory JDM patients despite the lack of evidence-based data and guidelines. Utilizing the nominal group techniques, we used this survey data and subsequent CARRA JDM study group meetings to suggest four potential biologic treatments for consideration for current use and future study. The next step may be to study the comparative effectiveness and safety of rituximab, abatacept, tocilizumab, and anti-TNF’s infliximab/adalimumab for refractory JDM.

## Additional file

Additional file 1: Appendix A. CARRA survey: Use of Biologics in Juvenile Dermatomyositis. **Appendix B** Second CARRA JDM Survey: Use of Biologics in refractory/resistant JDM patients. **Appendix C** Results of 2ND Biologic Survey of CARRA. (DOCX 65 kb)

## Abbreviations

AZA: azathioprine
CARRA: Children’s Arthritis and Rheumatology Research Alliance
CER: comparative effectiveness research
CSA: cyclosporine A
CTP: consensus treatment plan
IVIG: intravenous gamma globulin
JDM: juvenile dermatomyositis
MMF: mycophenolate
MTX: methotrexate
PR: pediatric rheumatologist
Steroids: corticosteroids
