# Supplementary material for: Biologic therapies for refractory juvenile dermatomyositis: five years of experience of the Childhood Arthritis and Rheumatology Research Alliance in North America
Source: Pediatr Rheumatol Online J. 2017 Jun 13;15:50. doi: 10.1186/s12969-017-0174-0 (PMC5470177; doi:10.1186/s12969-017-0174-0)
Supplement: Additional file 1: Appendix A. — CARRA survey: Use of Biologics in Juvenile Dermatomyositis. Appendix B Second CARRA JDM Survey: Use of Biologics in refractory/resistant JDM patients. Appendix C Results of 2ND Biologic Survey of CARRA. (DOCX 65 kb) [file 12969_2017_174_MOESM1_ESM.docx]

Additional file

**Appendix A**

CARRA survey: Use of Biologics in Juvenile Dermatomyositis

Name of the physician: Date:

Institution: Country: USA/Canada..................................

1. **About you:**

Years post fellowship:

Year of Fellowship:

Your special interest:

1. SLE
2. JIA
3. Vasculitis
4. IIM
5. Scleroderma
6. Basic science research
7. Nothing specific
8. Others

...........

Approximately how many total JDM patients currently do you see per year?

1. 1-10
2. 11-20
3. 20-50
4. 0ver 50

How many new JDM patients you see per year:

1. 1-10
2. 11-20
3. 20-50
4. 0ver 50
5. **Your practice and experience of using biologics in JDM.**

Have you used biologics in JDM?

1. Yes

No

Yes

- - 1. Etanercept

Yes

No

- - 1. Abatacept

Yes

No

- - 1. Adalimumab

No

Yes

- - 1. Etanercept

No

Yes

- - 1. Infliximab

No

No

Yes

- - 1. Anakinra

No

Yes

- - 1. Tocilizumab

Yes

No

- - 1. Rituximab

............................

- - 1. Others

1. No

No

Yes

1. If opportunity arises then will you use biologics agents in Juvenile DM

No

Yes

1. You did not use biologics agents in Juvenile DM because you do not believe it works
2. You did not use biologics agents in Juvenile DM because of insurance denial

No

Yes

No

Yes

No

1. You did not use biologics agents in Juvenile DM because of patients/parents denial.

Yes

1. You did not use biologics agents in Juvenile DM because you are not sure if it works

Yes

No

1. You did not use biologics agents in Juvenile DM because of cost of therapy

Why you did not use the biologics?

1. Did not have opportunity
2. I have reservation for use in Juvenile DM
3. I will use if opportunity arise
4. I am not sure it works well in Juvenile DM

What was the rationale to use biologics?

1. Uncontrolled disease
2. Steroid toxicity/MTX toxicity
3. Family request
4. Steroid dependent
5. Other

In which situation did you used biologics in Juvenile DM?

1. After failed MTX
2. After failed MTX+Steroids
3. After Failed MTX+Steroids+IVIG
4. After Failed MTX+Steroids+IVIG/AZA/MMF/CSA
5. For systemic Juvenile DM
6. For severe ulcerative disease
7. Others

.............

On how many patients have you used biologics so far?

1. 1-2
2. 3-5
3. 5-10
4. Over 10

Mostly you have used biologics:

1. As a monotherapy
2. In combination with MTX
3. In combination with MTX + steroids
4. In combination with MTX + steroid + and/or MMF/AZA/CSA
5. In combination with IVIG+MTX

.............

1. Others
2. **Outcomes:**

In your experience, the biologics agent led to:

1. Improvement
2. No change
3. Less calcinosis after use of biologics agent
4. Worsened
5. Unable to assess
6. Others

.............

Did your patient experienced toxicities?

Expandable text box for free text

1. Yes

Expandable text box for free text

1. No

Expandable text box for free text

1. Not sure
2. **Do you recommend that CARRA study any of these drugs for JDM**

Expandable text box for free text

1. Yes

Expandable text box for free text

1. No
2. **Your comments on use of biologics agents in JDM**

Expandable text box for free text

**Appendix B**

Second CARRA JDM Survey: Use of Biologics in refractory/resistant JDM patients

**A. Information to establish baseline current practice to develop CTP:**

**Q1:** In your opinion what should be the criteria that allow a patient to use these treatment plans (inclusion criteria)?

1. **…………………..**
2. **…………………….**
3. **…………………….**
4. **…………………….**
5. **……………………..**
6. ……………………….
7. ………………………..
8. ……………………….
9. ………………………
10. …………………………
11. ……………………….
12. …………………………….
13. ………………………………….
14. …………………………………

**Q 2:** In your opinion what should be the criteria that exclude a patient from using these treatment plans (exclusion criteria)?

1. …………………..
2. ……………………
3. ………………………
4. ……………………….
5. ……………………….
6. ………………………………………
7. ………………………………….
8. ………………………………….
9. …………………………………….
10. ………………………………………….
11. ……………………………………..
12. …………………………………..
13. ………………………………………..
14. ……………………………………….

**Q 3:** What elements of the initial work-up – reflecting current practice – should be standard for all included patients i.e. CBC, inflammatory markers, factor VIII-related antigen, muscle enzymes, RI, muscle biopsy, EMG, MSA?

1. ………………………
2. ………………………..
3. …………………………
4. ………………………….
5. ………………………….
6. ………………………….
7. …………………………

**Q 4:** What are the most important non-medicinal treatments – reflecting current practice – that should be used by included patients?

1. ………………
2. ………………..
3. ………………..
4. …………………..

**Q 5:** At what intervals should patients be followed for the purposes of data collection? Please distinguish data collection points from follow-up (i.e. Patients may be seen more frequently for their clinical needs)

1. …………………
2. …………………
3. …………………
4. …………………
5. ……………….

**Q6:** What elements of the follow-up work-up should be considered standard for all included patients?

1. ………………………
2. ………………………
3. ………………………
4. ……………………..
5. ………………………
6. ………………………
7. ………………………

**B.** It is believed that a select group of JDM patients may be candidates for biology therapy. In first survey two questions/concerns were raised. One about experience of using more than one biologic agent at a time and second, occasional flares presumed to be associated /caused by the use of biologics.

**Here we ask for little more on your experience:**

**Q 7:** Have you used more than one biologic agent at a time to treat JDM?

1. Yes
2. No

**Q 8:** If you will chose to use biologic agent in refractory JDM patients, then which will be your first and which will be your second choice. Please give the rationale for your selection. Please select only two agents and give dose and frequency also.

| Agent | First choice | Rationale for first choice | Second Choice | Rationale for second choice |
| --- | --- | --- | --- | --- |
| Rituximab |  |  |  |  |
| Abatacept |  |  |  |  |
| Etanercept |  |  |  |  |
| Infliximab |  |  |  |  |
| Adalimumab |  |  |  |  |
| Other |  |  |  |  |

**Q 9a:** Did one of your JDM patients ever experience a disease flare while on a biologic agent?

1. Yes
2. No

**Q 9b:** Did one of your JDM patients ever experience a disease flare likely related to use of biologics?

1. Yes
2. No

**Q 9d:** If your patient experienced disease flare with the use of biologics, then at what stage did it occur?

1. Immediately after start of the therapy with the biologic agent
2. After ………….weeks in the therapy with the biologic agent
3. After ………….months in the therapy with the biologic agent
4. Do not remember
5. Immediately after stopping the therapy with the biologic agent
6. ………..Weeks after stopping the therapy with the biologic agent

**Q9d:** Will you consider measuring Serum IFN alpha activity before starting anti TNF antibodies using a functional reporter cell assay to prevent drug induced flares?

**Q 10:** If you have used biologics in combinations in JDM, what other drugs were usually used with the biologic? Please describe different combinations you may have utilized.

1. ………………………..
2. ………………………..
3. ………………………..
4. ………………………..
5. ………………………..
6. ………………………..
7. ………………………..

We appreciate your attention and time. We will return to share the survey results with you soon.

Thanks

**APPENDIX C**

**Results of 2^ND^ Biologic Survey of CARRA**

Initial Screening Question: Do you care for children with JDM in your practice?

Results: Yes 115 (93.5%) No 8 (6.5%) Responders who say no ended the survey

Repeat of question in first survey-70 responders

Would you consider using a biologic agent in refractory JDM patients?

Yes=70/70 (100%)

Questions pertaining directly to use of biologics: Of a potential 107 variable number of respondents answered different parts of the survey

1. Have you used more than 1 biologic therapy in combination to treat JDM?

Yes 5 (7%) No 63 (93%)

1. TNF inhibitor (etanercept or infliximab) with rituximab
2. Il-6 inhibitor (tocilizumab) with rituximab
3. Infliximab and rituximab
4. If you have used biologics in combination with other non-biologics in JDM, what other drugs were usually used with the biologic. Please describe different combinations you may have utilized and the rationales of their use.
5. Methotrexate + infliximab

Rationale: better efficacy in arthritis

1. Methotrexate + CSA,+ IVIG + prednisone with a biologic

Rationale: Continued rash, elevated muscle enzymes, steroid dependency

1. Biologic in combination with methotrexate or in combination with MMF

Rationale: “Bad cases”

1. MTX/MMF/AZA/CSA + steroids + biologic.

Rationale: “I really do not have a strong reason-empirical use”

1. Methylprednisolone + CSA + IVIG + Plaquenil + biologic

Rationale: None given

1. Cyclophosphamide + abatacept

Rationale: None given

1. Methotrexate + cyclophosphamide + IVIG + rituximab

Rationale: In a patient with persistent respiratory muscle weakness

1. Methotrexate + steroids + IVIG + etanercept

Rationale: Prominent synovitis, persistent muscle enzyme elevation, elevated vWF, persistent proximal muscle weakness

1. Tacrolimus + Mycophenolate + Mtx + IVIG + pamidronate in combination with one of 5 biologics: abatacept, anakinra, etanercept, infliximab, or rituximab.

Rationale: None given

1. Abatacept + cyclosporine + IVIG.

Rationale: for the treatment of severe active disease and calcinosis

1. Steroids + methotrexate + biologic

Rationale: for severe arthritis with JDM

1. Steroids + hydroxychloroquine + methotrexate + infliximab

Rationale: tenosynovitis

1. Methylprednisolone pulsing + a biologic

Rationale: none given

1. IVIG + infliximab

Rationale: different mechanisms of action

1. Tacrolimus + biologic

Rationale: for ulcerating disease

1. Hydroxychloroquine + biologic

Rationale: persistent skin disease

1. TNF biologics

Rationale: ulcerative disease

1. TNF biologics

Rationale: Usually used in patient with recalcitrant disease-not improving, needing steroids,

and with arthritis, too.

1. Use rituximab with methotrexate and steroids and if no response, continue methotrexate and steroids and add abatacept

Rationale: none given

1. Diltiazem or other calcium channel blocker or bisphosphonate, in combination with biologic

Rationale: Calcinosis

1. Rituximab + oral cyclophosphamide

Rationale: 1) In a patient who failed prednisone , methotrexate, IVIG, and mycophenolate; 2) for purpose of decreasing HACA formation and increase drug synergy of effect

3. If you would consider using a biologic agent in refractory JDM patients, then which will be your first and which will be your second choice (i.e., first and second mono therapy biologics)? Please give a rationale for your selection in the comment section. Please only select two agents and dose and frequency also:

Results:

1. Rituximab

Primary biologic-48/66 (73%) Secondary biologic 9/55 (16%)

1. Abatacept

Primary biologic-7/66 (10%) Secondary biologic 16/55 (29%)

1. Etanercept

Primary biologic-3/66 (4%) Secondary biologic 6/55 (11%)

1. Infliximab

Primary biologic-7/66 (10%) Secondary biologic 16/55 (29%)

1. Adalimumab

Primary biologic-1/66 (2%) Secondary biologic 5/55 (9%)

1. Other-Tocilizumab x 2, ustekinumab x 1

-Primary biologic-0/66 Secondary biologic 3/55 (6%)

Note: Not every responder picked a secondary biologic-only 55

Rationales given for use of a biologic as a primary biologic

1.Rituximab

a. Based on RIM study and open label data published

b. But for rituximab use the patient has to be stable and the response is quite delayed, so have to treat with interim therapy until it kicks in.

c. Excellent success rate for inducing durable remission, more rapid response and higher long-term success rate than IVIG, and low rates of complications, good compliance rates and patient acceptance..

d. I think it makes more sense to work on T & B cells rather than TNF.

e. Perhaps mechanism of efficacy is similar to IVIG-inhibitory to fc receptors on monocytes/macrophages/dendritic cells

f. Rituximab has been steroid-sparing for us; it is a great drug for vasculitis, lupus, ILD.

g. Comfort level and data

h. In muscle predominant resistant disease I will use rituximab to influence autoantibodiy production against endothelial cells.

i. Rituximab is useful in other vasculitides so its use makes biological sense in JDM

2. Anti-TNF medications

a. In ulcerative disease I would like to use anti-TNF medications to influence TNF which is more likely to be a mediator.

b. Etanercept washes out sooner than the other TNF-alpha inhibitors so it is my first choice if there is no uveitis or other compelling reason to use either an IV or longer-acting agent such as adalimmab.

c. More data on TNF inhibitors although the data on treating children with myositis with rituximab looks promising

d. Good results with ulcerative skin lesions

e. Patients with calcinosis based on London GOSH experience

f. More anecdotal experience and case reports than other biologics

g. TNF polymorphisms have been associated with disease based on case reports and anecdotal evidence.

3. Abatacept

a. Proinflammatory cytokines induce expression of CTAL4 and CD28 on muscle cells; 2012 case report suggests efficacy in a patient with an overlap syndrome with myositis who was rituximab resistant. b. I think it makes more sense to work on T & B cells rather than TNF. c. Abatacept has a unique mechanism of action of biologics and also has a pretty broad range of use in in rheumatic disorders. d. It seems like a CD4 mediated disease from a pathological standpoint e. Comfort level and data.

4. Other (tocilizumab and ustekinumab)

a. Tocilizumab

1) I am curious about the use of IL-6 blockade in refractory JDM. It appears in some case reports with good responses

2) Based upon on some of the work on the new autoinflammatory syndromes (CANDLE, etc.) with elevated IL-6 signature

3) It seems to improve strength more quickly as compared to the anti-TNF biologics. However I do see more cytopenias and URI’s.

b. Ustekinumab

1) It is being tried in psoriasis and we will see how that goes.
